# Multi-Factor Analysis of Single-Center Asthma Control in Xiamen, China

**DOI:** 10.3389/fped.2019.00498

**Published:** 2019-12-03

**Authors:** Yu Zhu, Taoling Zhong, Dandan Ge, Qiyuan Li, Jinzhun Wu

**Affiliations:** ^1^Department of Pediatrics, The First Affiliated Hospital of Xiamen University, Xiamen, China; ^2^National Institute for Data Science in Health and Medicine, School of Medicine, Xiamen University, Xiamen, China

**Keywords:** childhood asthma, disease control rate, risk factors, allergic history, air quality

## Abstract

We evaluated the effects of air pollutants, age, allergic history, family allergic history, treatment, treatment steps, and compliance on uncontrolled childhood asthma in Xiamen, China. The clinical data of children with asthma in the pediatric outpatient department of the First Affiliated Hospital of Xiamen University from January 2016 to June 2018 were analyzed retrospectively. According to the assessment of the patients' outcome including well-controlled, partly-controlled and uncontrolled, 7,211 cases of 3,268 patients were selected. Rank sum test and ordered multi-class logistic regression analysis were used. In the rank sum test, age, allergic history, family allergic history, season, treatment, treatment steps and compliance were found associated with uncontrolled rate (all *P* < 0.001). Logistic regression analysis showed that PM_10_, NO_2_, and SO_2_ raised uncontrolled-asthma rate (aOR 1.311, aOR 1.281, aOR 1.252, respectively). Older children had lower uncontrolled rate (OR = 0.849, 95% CI: 0.758–0.950), children with higher treatment steps had higher uncontrolled rate (OR = 1.227, 95%CI: 1.040–1.448), and children with better treatment compliance have lower uncontrolled rate (OR = 0.374 95% CI: 0.331–0.424). The order of the uncontrolled rate of asthma from high to low was winter, spring, autumn, and summer. PM_10_, NO_2_, SO_2_, age, season, treatment steps, and treatment compliance have significance for predicting the control rate of childhood asthma in Xiamen, China.

## Introduction

Asthma is a common chronic respiratory disease, especially in children. Epidemiological study found that the prevalence of asthma is increasing year by year (1, 2). The control of asthma is closely related to the quality of life of patients.

Children's asthma is related to various factors such as environmental factors, genetic factors, and immunological factors. Environmental pollution plays a part in asthma exacerbation, and there are a few investigations regarding to the relationship between air pollutants and childhood asthma in China. PM_1_, PM_2.5_, and PM_10_ exposure had a negative effect on adolescent respiratory system and was associated with asthma exacerbation (3, 4). NO_2_, SO_2_, and CO were risk factors of severe childhood asthma (5–7). However, the research on the air pollution and childhood asthma control is rare in China.

The control of asthma is far from encouraging. In one worldwide survey, the prevalence of severe asthma symptoms (four or more attacks of wheeze, waking at night with asthma symptoms one or more times per week, and/or any episodes of wheeze severe enough to limit the ability to speak) was more than 7.5% in numerous centers in the UK, New Zealand, Brazil and other countries in the preceding 12 months (8). Asthma prevalence varies from 1 to 18% in different part of the world, with the highest prevalence (6.0–12.0%) in developed western countries (9). A global investigation presented that the hospitalized asthma patients are decreasing (8), which indicated that outpatient management of asthma is important. We found some recent studies from China on associations between personal statements and children asthma exacerbation but a few studies on childhood asthma control. Xiang's study found that the risk of uncontrolled asthma was higher in subjects with treatment non-adherence, concomitant allergic rhinitis (AR), disease duration ≥ 1.5 years, and first-degree relatives with AR (10). One study from Beijing found that children with asthma showed significant improvements in control rates and lung function during control-based asthma management (11). Thus, there is a need for more studies on asthma control among children in China in relation to personal statements.

In order to better guide the patients and their family in developing countries to cooperate with doctors, the risk factors of the decreasing asthma control rate in Xiamen were studied. We obtained real-world data of childhood asthma cases from the electronic medical record system of the First Affiliated Hospital of Xiamen University to provide theoretical support for asthma management.

## Methods

### Clinical Data

The data of pediatric respiratory diseases from January 1, 2016 to June 30, 2018 were obtained from the pediatric outpatient electronic medical record system of the First Affiliated Hospital of Xiamen University. The data were from urgent care, department of asthma and outpatient. The patients were followed up regularly (every one, 2 or 3 months). At the follow-up, the doctors who work in the First Affiliated Hospital of Xiamen University recorded the patient's condition and judged the control status according to examination, diurnal and nocturnal symptoms. The data included the visit date, the patient ID, the patient's name, age, gender, chief complaint, current medical history, diagnosis, family history, past medical history, etc. All patients were aged between 0 and 14 years old.

According to the “Guidelines for the Diagnosis and Prevention of Childhood Bronchial Asthma” developed by the Department of Respiratory Medicine of the Chinese Medical Association in 2016 (12), 17,835 childhood asthma cases (J45 in ICD10) were selected from the diagnosis results of the medical records (Supplementary Table 1). According to the symptoms and examinations 4 weeks before the patients' visit, the patient's outcome was evaluated as “well-controlled,” “partly-controlled,” and “uncontrolled” (Supplementary Table 2). The treatment was decided based on patients' outcomes (Supplementary Figures 1, 2). After removal 175 patients from other cities, a total of 7,211 cases of 3,268 patients were enrolled. And 35 patients were asked to be admitted after the outpatient visits.

### Air Pollution Data

The data of air pollution including PM_2.5_, PM_10_, CO, SO_2_, NO_2_ were collected at four different sites in the city, with the help of Xiamen Department of Environmental Protection. The concentration of pollutants was measured and we took the average of every site. Meteorological data, such as temperature, wind speed, precipitation and so on, were got from Xiamen Meteorological Bureau. March, April, and May are defined as spring, June, July, and August are summer, September, October, and November are autumn, and December to February are winter.

### Statistical Analysis

We used Wilcoxon and Kruskal-Wallis rank sum test to investigate the relationship between control levels and the personal statements containing gender, season, age, family allergic history, allergic history, treatment, and compliance. The variables which showed significant differences were included in multiple logistic regression analysis. The average concentration of air pollutants, including PM_2.5_, PM_10_, CO, NO_2_, and SO_2_, in the 4 weeks before the patient's visit was included in the ordered multi-class logistic regression analysis. We marked the “well-controlled asthma” as 1, the “partly controlled asthma” as 2 and “uncontrolled asthma” as 3. In order to eliminate the difference of air pollutants, we divided the pollutant concentration by the interquartile range (IQR) to get the adjusted OR (aOR). Statistical significance was set at *p* < 0.05. Data filter and statistical analysis were conducted in R 3.5 software with the “MASS” package.

## Results

### Summary of Air Pollutants

**Figure 1:**
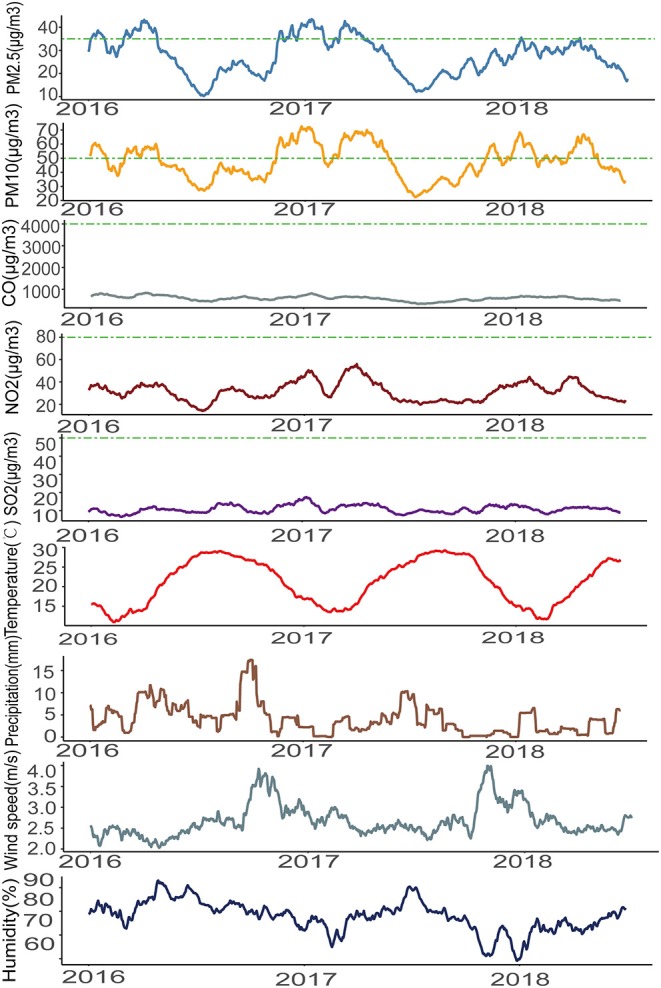
The air pollutants and the meteorological data (mean values of the previous 4 weeks of the visits) during the study period.

We calculated the average of the pollutant concentration and meteorological data in 4 weeks before the visits. The average concentration of PM_2.5_ was 27.25 μg/m^3^, the minimum was 10.29 μg/m^3^, and the maximum was 43.50 μg/m^3^. The PM_10_ concentration range was 22.39–72.61 μg/m^3^ and the annual mean was 47.50 μg/m^3^. The average concentration of CO was 584.70 μg/m^3^ and the concentration ranged from 332.10 μg/m^3^ to 839.30 μg/m^3^. The average of NO_2_ was 31.48 μg/m^3^, and the median was 31.29 μg/m^3^. The mean of SO_2_ was 10.84 μg/m^3^ and the median was 10.64 μg/m^3^ (Table 1). The study period contains 912 days. According to “Technical Regulation on Ambient Air Quality Index” (13), the standard of PM_2.5_ is 35 μg/m^3^ and there were 198 days whose average concentration in the previous 4 weeks exceed the standard; the standard of PM_10_ is 50 μg/m^3^ and in 43.09% days the concentration of PM_10_ exceeds the standard; the standard of CO is 4,000 μg/m^3^, the concentration of NO_2_ is 80 μg/m^3^ and the concentration of SO_2_ is 50 μg/m^3^, and all the average concentration was below the standard of “Rank 1” (Figure 1).

**Table 1:** Summary of air pollutants and environmental variables.

	**Mean**	**Standard deviation**	**Minimum**	**First quartile**	**Median**	**Third quartile**	**Maximum**
PM_2.5_ (μg/m^3^)	27.25	8.23	10.29	21.07	27.25	33.93	43.50
PM_10_ (μg/m^3^)	47.50	12.33	22.39	38.96	46.14	57.93	72.61
CO (μg/m^3^)	584.70	111.00	332.10	507.10	589.30	653.60	839.30
NO_2_ (μg/m^3^)	31.48	8.71	14.04	23.82	31.29	37.21	56.00
SO_2_ (μg/m^3^)	10.84	2.03	6.57	9.21	10.64	12.36	17.29
Temperature (°C)	21.52	5.80	10.97	15.63	22.51	27.01	29.25
Precipitation (mm)	4.00	3.29	0.01	1.48	3.48	5.43	17.39
Wind speed (m/s)	2.69	0.39	2.03	2.44	2.57	2.80	3.99
Humidity (%)	77.87	6.63	59.14	73.75	78.50	81.96	93.00

### The Associations Between Personal Statements and the Asthma Control

**Figure 2:**
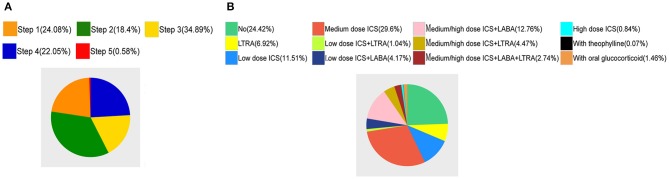
**(A)** The Pie chart of the treatment steps of the patient. **(B)** The Pie chart of the medication prescribed.

There were 1,453 patients who were in step 1, 1,119 in step 2, 2,105 in step 3, 1,330 in step 4, and 35 in step 5 (Figure 2A). In 5,950 visits with complete prescription data, 412 people used leukotriene receptor antagonist (LTRA) alone and 1,453 patients had no medication prescribed. The medication prescribed was shown in Figure 2B. Among the enrolled visits, 4,815 (66.77%) visits were male patients and 2,396 (33.23%) were female patients. The prevalence of asthma in males was higher. The well-controlled rate was 41.20% for males and 41.19% for females. The uncontrolled rate of male patients was 12.88% while the uncontrolled rate of female patients was 12.10%. The gender difference in control was not significant (*P* = 0.763). According to the age, the patients were divided into three groups: infant (0–3 years old), preschool children (4–6 years old), and school-aged children (7–14 years old). There were 3,412 children in preschool group, accounting for the largest proportion (47.3%), which suggests that preschool children are most susceptible to asthma. Table 2 showed that there were differences in the control rate of the three age groups (*P* < 0.001).

**Table 2:** Summary of gender, age, allergic history, allergic family history, visit seasons, treatment and compliance, and the assessment of relationship with asthma control from rank sum test.

	**Total**	**Well-controlled**		**Partly-controlled**		**Uncontrolled**			***P*-value**
		***N***	**%**	***N***	**%**	***N***	**%**		
**Gender**								*W* = 5,791,200	0.763
Male	4,815	1,984	41.20%	2,211	45.92%	620	12.88%		
Female	2,396	987	41.19%	1,119	46.70%	290	12.10%		
**Age (years)**								*H* = 145.64	<0.001^***^
0–3	1,869	632	33.81%	919	49.17%	318	17.01%		
4–6	3,412	1,347	39.48%	1,640	48.07%	425	12.46%		
7–14	1,930	992	51.40%	771	39.95%	167	8.65%		
**Allergic history**								*W* = 4,523,700	<0.001^***^
With	3,797	1,617	42.59%	1,700	44.77%	480	12.64%		
Without	2,524	947	37.52%	1,205	47.74%	372	14.74%		
Missing	890								
**Allergic family history**								*W* = 3,304,100	<0.001^***^
With	1,895	881	46.49%	803	42.37%	211	11.13%		
Without	3,858	1,466	38.00%	1,814	47.02%	578	14.98%		
Missing	1458								
**Season**								*H* = 109.67	<0.001^***^
Spring	2,056	830	40.37%	893	43.43%	333	16.20%		
Summer	1,911	893	46.73%	843	44.11%	175	9.16%		
Autumn	1,425	650	45.61%	651	45.68%	124	8.70%		
Winter	1,819	598	32.88%	943	51.84%	278	15.28%		
**Treatment**								*H* = 138.53	<0.001^***^
ICS	4,444	2,161	48.63%	1,829	41.16%	454	10.22%		
LABA	824	472	57.28%	296	35.92%	56	6.80%		
LTRA	964	301	31.22%	526	54.56%	137	14.21%		
Theophylline	4	1	25.00%	1	25.00%	2	50.00%		
Oral corticosteroid	87	37	42.53%	34	39.08%	16	18.39%		
Missing	901								
**Steps**								*H* = 116.79	<0.001^***^
Step 1	1,453	455	31.31%	779	53.61%	219	15.07%		
Step 2	1,119	487	43.52%	523	46.74%	109	9.74%		
Step 3	2,105	1,057	50.21%	811	38.53%	237	11.26%		
Step 4	1,330	584	43.91%	608	45.71%	138	10.38%		
Step 5	35	12	34.29%	17	48.57%	6	17.14%		
Missing	1,169								
**Treatment compliance**								*H* = 549.7	<0.001^***^
Regular medication	3,423	2,265	66.17%	901	26.32%	257	7.51%		
Irregular medication	397	79	19.90%	233	58.69%	85	21.41%		
No medication	340	59	17.35%	215	63.24%	66	19.41%		
Missing	3,051								

*** *P < 0.001*.

Among the 6,321 patients with a history record, 3,797 had a history of allergic disease accounting for 60.07%, and 2,524 had no history of allergic disease (Table 2). There was a difference in the control rate between the two groups with or without allergic disease (*P* < 0.001). Among the 5,753 patients with a family history record, there were 1,895 (32.94%) patients whose three-generation relatives had allergic diseases, and 3,858 (67.06%) patients did not have family allergic history. There was a significant difference between the two groups with or without family allergic history (*P* < 0.001).

In 7,211 visits, 2,056 visits were in spring, 1,911 in summer, 1,425 in autumn and 1,819 in winter. There were 893 well-controlled visits in summer, accounting for 46.73% of the visits in summer but there were only 32.88% well-controlled visits in winter. The Kruskal-Wallis rank sum test showed that there were differences in the control of the four groups (Table 2).

We analyzed every drug separately including ICS, long-acting beta_2_-agonist (LABA), LTRA, theophylline and oral corticosteroid. Among the 6,320 patients with prescription data, 4,444 patients used ICS, and the well-controlled rate was 48.63%. Nine hundred and sixty-four patients had LTRA and the well-controlled rate was 31.22%. The Kruskal-Wallis rank sum test showed that the control of using various drugs were different. The difference of control rates among five steps was statistically significant (*P* < 0.001). Therefore, we included treatment steps in the logistic regression. Among the 4,160 cases recorded with treatment compliance (regular medication, irregular medication, no medication), 3,423 (82.28%) patients had regular medication and the well-controlled rate was 66.17%. There were 340 (8.17%) patients who did not use the drug prescribed, among whom the well-controlled rate was 17.35%. The difference of distribution of control levels among the three groups was significant (*P* < 0.001).

### Logistic Regression Analysis of Multiple Factors

The effects of PM_2.5_, PM_10_, CO, NO_2_, and SO_2_ on asthma control were adjusted by the average temperature, precipitation, wind speed and humidity, respectively. Table 3 indicated that the adjusted OR. The aOR of PM_10_ was 1.311 (95% CI 1.185–1.449). The aOR of NO_2_ was 1.281 (*P* < 0.001). The SO_2_ augmented the uncontrolled rate (aOR 1.252, 95% CI 1.166–1.344). But PM_2.5_ concentration was not relative to the asthma control rate (*P* = 0.273), and the influence of CO on asthma control had no statistical significance either.

**Table 3:** Results of aORs and 95% CIs from ordered multi-class logistic regression.

**Variables**	**aOR**	**95% CI**		***P*-value**
PM_2.5_ (μg/m^3^)	1.069	0.949	1.205	0.273
PM_10_ (μg/m^3^)	1.311	1.185	1.449	<0.001^**^
CO	1.098	0.9991	1.207	0.051
NO_2_	1.281	1.165	1.408	<0.001^**^
SO_2_	1.252	1.166	1.344	<0.001^**^

** *P < 0.01*.

Of 7,211 cases, 3,026 cases which had records of age, allergic history, family allergic history, visit date, treatment, steps, and compliance were analyzed by ordered multi-class logistic regression. The results were shown in Table 4. Age was associated with control and older children had lower asthma uncontrolled rate (OR 0.849, 95% CI 0.758–0.950, *P* = 0.004). The seasons affected childhood asthma control rate (OR 0.818 95% CI 0.768–0.872 *P* < 0.001). In summer, the control was best. The associations between treatment steps and asthma control remained statistically significant (OR 1.227, 95% CI 1.040–1.448, *P* = 0.015).Treatment adherence (OR 0.374, 95% CI 0.331–0.424, *P* < 0.001) was a risk factor in uncontrolled childhood asthma. The drugs the patients used had no effect on the control.

**Table 4:** Association between asthma control and multiple factors among children in Xiamen.

**Variables**	**OR**	**95% CI**		***P*-value**
Age	0.849	0.758	0.950	0.004^**^
Allergic history	1.090	0.934	1.273	0.273
Family allergic history	1.013	0.872	1.175	0.869
Season	0.818	0.768	0.872	<0.001^***^
ICS	0.851	0.609	1.191	0.347
LABA	0.752	0.561	1.007	0.055
LTRA	1.075	0.813	1.422	0.610
Theophylline	3.484	0.182	66.858	0.417
Oral corticosteroid	0.947	0.478	1.878	0.876
Steps	1.227	1.040	1.448	0.015^*^
Treatment compliance	0.374	0.331	0.424	<0.001^***^

*We assessed the effect of personal statements on asthma control. ORs, 95%CIs and P-values are presented. ICS, inhaled corticosteroid; LABA, long-acting beta_2_-agonist; LTRA, leukotriene receptor antagonist*.

* P < 0.05,

** P < 0.01,

*** *P < 0.001*.

## Discussion

The diagnosis and control assessment of childhood asthma in this study were from Chinese guidelines which was made based on GINA (14). The Chinese doctors, especially in less-developed areas, use stethoscopes frequently, so the description of auscultation is more detailed in Chinese guidelines.

This study showed that, in the 7,211 visits in pediatric outpatients from 2016 to 2018, 2,971 cases were well-controlled, accounting for 41.20, 46.18% were partly-controlled and 12.62% were uncontrolled asthma. Thus, children with asthma in Xiamen are mostly partly-controlled. The asthma control rate in Europe is 55% (15). The US survey found that 55.8% of patients with had at least one asthma attack in the past year (16). In China, 60.0–64.9% of adults with asthma were uncontrolled (17, 18). The rate of uncontrolled childhood asthma in China needs a population-based study. It is suggested that the asthma control situation in Xiamen is consistent with that in other parts of China, but the asthma controlled rate in China is lower than that in developed countries.

We eliminated the effect of other weather conditions such as temperature, wind speed, etc. and concluded that PM_10_, NO_2_, and SO_2_ have a negative effect on childhood asthma control. The association between air pollution and asthma has been studied in different parts of the world. A study from South Africa showed that exposure to PM_10_, SO_2_, NO_2_, and NO is associated with significantly increased occurrence of respiratory symptoms among children (19). A study, in Bradford England, used a newly developed full-chain model to show that 38% of childhood asthma were caused by air pollution, and 6% of which attributed to NO_2_ and NO_x_ (20). This article provides a detailed explanation of the relationship between asthma control and air pollution within 4 weeks prior to the visits. We collected the air pollution data from four sites in Xiamen but the data did not reflect the exact living environment of the patients.

This study found that there was no effect of PM_2.5_ on the childhood asthma control. This contrasts with the results from several previous studies that have demonstrated the adverse effect of PM_2.5_ on asthma in children. A review summarized that children exposure to both PM_2.5_ and PM_10_ was associated with the aggravation of asthma symptoms (21). Each increase of 10 μg/m^3^ in concentrations of PM_2.5_ was associated with 1–2% increase in risk of wheeze-associated disorders (22). In particular, an earlier study conducted among school children in California found each 8.1 μg/m^3^ decrease in particulate matter <2.5 μm was associated with a reduction of 1.53 cases per 100 person-years in asthma incidence in their study while examine whether decreasing regional air pollutants were associated with reduced incidence of childhood asthma (23). However, several previous studies also failed to find an association between ambient PM_2.5_ and asthma in children. Mazenq who performed a study of the effects of air pollution on children in southeastern France, suggested that PM_10_ increased the risk of emergency asthma-related hospital visits, but PM_2.5_ were not found to be a risk factor (24). Other epidemiological survey also reported that PM_10_ is associated with asthma, but not PM_2.5_ (25). This might be due to the different composition of PM_2.5_ in different regions.

The Rank Sum Test of age, gender, allergic history, family allergic history, season, treatment, steps, and compliance showed that age, allergic history, family allergic history, season, treatment, steps, and compliance were related to asthma control. And these related factors were included into the logistic regression model. Multi-factor logical regression analysis showed that age, season and treatment steps and compliance were the risk factors of asthma control. The control rate increased with age and the highest control rate presented in children aged 7–14. With the increase of age, children's medication compliance is good, especially using aerosol therapy. They can make full use of the inhaled drugs. And children's immune system is constantly improving (26), which maintains immune balance and alleviates the Th1/Th2 cell imbalance, so that asthma symptoms are relieved (27, 28). Some studies reported that asthma control is related to the season, temperature, humidity and pollen (29–33). The control rate was different in different seasons, but the data of pollen lacked in this study. The impact of pollen and childhood asthma control requires further research. It is a common view that passive smoking is detrimental to respiratory system (34, 35), but there was no data of passive smoking in the study. In our study, treatment compliance had the greatest impact on asthma control. The Global Initiative for Asthma (GINA) guidelines (36) pointed out that management of ICS treatment is an influential factor in asthma control, suggesting that children should pay attention to the medication. In a systematic review, daily ICS appears more effective than daily LTRA for improving symptom control and decreasing exacerbation, when a minimum of 3-month therapy with daily ICS or LTRA was performed (37). However, in this study, the effect of varies of drugs on asthma control was not significant because only 4-weeks medication was observed. It is worth noting that compliance with medical advice and regular use of drugs can alleviate asthma symptoms and greatly improve the quality of life.

Bao et al., Bednarek et al., and Ahmadizar et al. found that allergic history and family history of allergies can predict asthma control rate (38–40). In this article, we studied whether allergic history and family allergic history were related to childhood asthma control, but the two characteristics were not risk factors. This happened because of the good economic conditions of patients who have the standardized management of asthma in Xiamen. Although these children have genetic susceptibility, their family pay more attention to collecting information and understand ways to prevent and treat asthma symptoms. We analyzed the relationship between allergic history, family allergic history and medication compliance, and found that these patients had more regular medication. Moreover, we found in the study that patients with a family allergic history have a higher rate of testing allergens. These patients and their family avoid contacting with allergens and pay more attention to home cleanness.

Ordered multi-class logistic regression was used to analyze risk factors of asthma control. Seven variables, including PM_10_, NO_2_, SO_2_, age, season, treatment steps, and compliance, were selected. These factors play an important role in the control and management of childhood asthma in Xiamen.

## Data Availability Statement

The data analyzed for this study can be found from the corresponding authors on reasonable request.

## Ethics Statement

This study was approved by the ethical guidance (KY2015–027) of the Ethical Review Board of the First Affiliated Hospital of Xiamen University. As private information including patient ID, residence and contact information is crypted and hashed, the review board agreed to waive the statement of consent.

## Author Contributions

YZ, JW, and DG acquired the data. YZ analyzed the data and drafted the manuscript. JW and QL revised the manuscript. All authors contributed to conception and design of the research, interpretation of the results, and edited the manuscript.

### Conflict of Interest

The authors declare that the research was conducted in the absence of any commercial or financial relationships that could be construed as a potential conflict of interest.
