# Supplementary material for: Multi-Factor Analysis of Single-Center Asthma Control in Xiamen, China
Source: Front Pediatr. 2019 Dec 3;7:498. doi: 10.3389/fped.2019.00498 (PMC6901658; doi:10.3389/fped.2019.00498)
Supplement: Supplementary file 1 [file Table_1.DOCX]

**Supplementary Table 1.** The asthma diagnosis criteria in the study (1).

| Asthma Diagnosis Criteria | |
| --- | --- |
| 1.Repeated onset of wheezing, cough, shortness of breath, chest tightness, and more related to contact with allergens, cold air, physical, chemical stimuli, respiratory infections, exercise and hyperventilation (such as laughter and crying), often at night and (or) after sleep. | Can be diagnosed as asthma if it meets 1-4 or, 4 and 5 |
| 2. During the exacerbation, sporadic or diffuse wheezing can be heard on auscultation especially on expiration, and the expiration is prolonged. |  |
| 3. The symptoms and signs relieve automatically or by treatment. |  |
| 4. Exclude wheezing, coughing, shortness of breath and chest tightness caused by other diseases. |  |
| 5. The patients with atypical clinical presentation (such as no wheezing) should have at least one of the following points: (1)the presence of reversible airflow limitation: 1) positive bronchodilator reversibility test: increase in forced expiratory volume in 1 second (FEV1)≥12%, 15 minutes after a short-acting beta_2_-agonist; 2) increase of lung function after anti-inflammatory treatment: increase in FEV1 by ≥12% after treatment with inhaled corticosteroids and/or leukotriene receptor antagonist for 4-8 weeks; (2) bronchial provocation test is positive; (3) average daily peak expiratory flow (PEF) variability ≥13% over 2 weeks. |  |

1. The Subspecialty Group of Respirology. The Society of Pediatrics, Chinese Medical Association guidelines for the diagnosis and prevention of asthma in children (2016). Chin. J Pediatr. (2016). <https://doi.org/10.3760/ema.j.issn.0578—1310.2016.03.003>.
