# Supplementary material for: Multi-Factor Analysis of Single-Center Asthma Control in Xiamen, China
Source: Front Pediatr. 2019 Dec 3;7:498. doi: 10.3389/fped.2019.00498 (PMC6901658; doi:10.3389/fped.2019.00498)
Supplement: Supplementary file 2 [file Table_2.DOCX]

**Supplementary Table 2.** Assessment of disease control of asthma for children below and above 6 years old (1).

| Children aged 6 years or older |  |
| --- | --- |
| 1. Daytime symptoms more than twice/week | None of the items is classified as well controlled asthma; 1-2 items as partly controlled asthma; 3-4 items uncontrolled asthma |
| 2. Night waking due to asthma |  |
| 3. Reliever needed for emergency more than twice/week |  |
| 4. Activity limitation due to asthma |  |
| Children under 6 years old |  |
| 1. Daytime symptoms lasting at least several minutes more than once/week | None of the items is classified as well controlled asthma; 1-2 items as partly controlled asthma; 3-4 items uncontrolled asthma |
| 2. Night waking or cough due to asthma |  |
| 3. Reliever needed for emergency more than once/week |  |
| 4. Activity limitation (less running/playing compared to other children, easy to fatigue when walking/playing) due to asthma |  |

1. The Subspecialty Group of Respirology. The Society of Pediatrics, Chinese Medical Association Guidelines for the diagnosis and prevention of asthma in children (2016). Chin. J. Pediatr. 2016. doi:10.3760/ema.j.issn.0578—1310.2016.03.003
