# Supplementary material for: Multi-Factor Analysis of Single-Center Asthma Control in Xiamen, China
Source: Front Pediatr. 2019 Dec 3;7:498. doi: 10.3389/fped.2019.00498 (PMC6901658; doi:10.3389/fped.2019.00498)
Supplement: Supplementary file 3 [file Data_Sheet_1.docx]

Supplementary Material


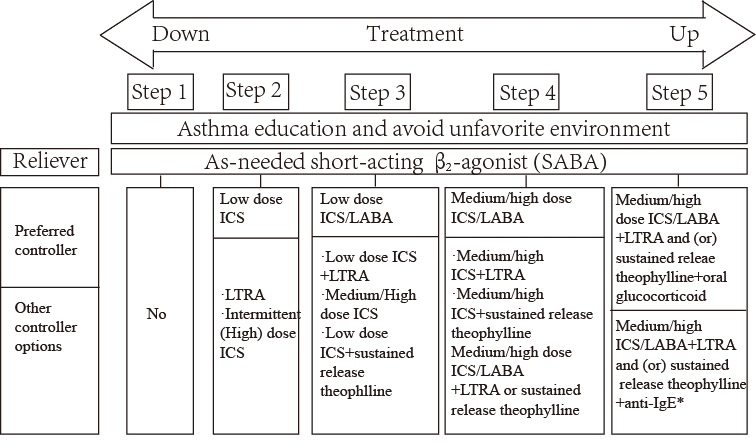


ICS: inhaled corticosteroid; LTRA: Leukotriene receptor antagonists; LABA: Long-acting β2 agonists; *anti-IgE is used for children ≥6 years old

**Supplementary Figure 1.** Long-term treatment of asthma in children older than or equal to 6 years old.


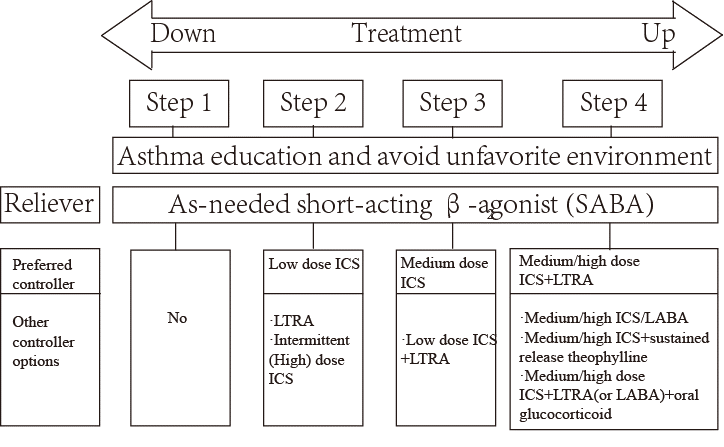


ICS: inhaled corticosteroid; LTRA: Leukotriene receptor antagonists; LABA: Long-acting β2 agonists

**Supplementary Figure 2.** Long-term treatment of asthma in children younger than 6 years old.
